# Supplementary material for: Self-Reported Cannabis Use and HIV Viral Control among Patients with HIV Engaged in Care: Results from a National Cohort Study
Source: Int J Environ Res Public Health. 2022 May 6;19(9):5649. doi: 10.3390/ijerph19095649 (PMC9101884; doi:10.3390/ijerph19095649)
Supplement: Supplementary file 1 [file ijerph-19-05649-s001.zip › ijerph-1635047-supplementary.pdf]

Supplementary Materials

Table S1. Sociodemographic and clinical characteristics associated with cannabis use categories among people living with HIV receiving antiretroviral therapy in the VACS cohort (2002–2018).

Characteristic	Overall (N= 2515)	No lifetime use (N=737, 29.3%)	Lifetime but none in past year (N=1093, 43.5%)	Past year use (N=685, 27.2%)	Global *p*-value
**Demographics**					
**Age, mean (SD)**	50.1 (8.8)	51.2 (10.6)	50.2 (7.7)	48.7 (8.2)	<0.0001
**Gender, *n* (%)**					0.762
Male	2447 (97.3)	719 (97.6)	1064 (97.4)	664 (96.9)	
Female	68 (2.7)	18 (2.4)	29 (2.7)	21 (3.1)	
**Race/ethnicity, *n* (%)**					0.133
Non-Hispanic White	524 (20.8)	138 (18.5)	227 (20.8)	161 (23.5)	
Non-Hispanic Black	1646 (65.5)	500 (67.8)	714 (65.3)	432 (63.1)	
Hispanic (any race)	247 (9.8)	80 (10.9)	105 (9.6)	62 (9.1)	
Other (multiple race or unknown)	98 (3.9)	21 (2.9)	47 (4.3)	30 (4.4)	
**Education, *n* (%)**					0.205
High school or less	987 (39.7)	297 (41.0)	439 (40.6)	251 (36.9)	
Some college or more	1500 (60.3)	427 (59.0)	643 (59.4)	430 (63.1)	
**Marital status, *n* (%)**					0.164
Never married	610 (24.6)	186 (25.7)	254 (23.6)	170 (25.2)	
Married/living with a partner	981 (39.6)	278 (38.4)	456 (42.3)	247 (36.7)	
Divorced/widowed	885 (35.7)	260 (35.9)	368 (34.1)	257 (38.1)	
**Housing instability ever, *n* (%)**	982 (39.3)	192 (26.2)	488 (44.9)	302 (44.3)	<0.0001
**Location of residence, *n* (%)**					0.670
Urban	2368 (95.0)	691 (94.8)	1035 (95.5)	642 (94.3)	
Suburban	70 (2.8)	20 (2.7)	30 (2.8)	20 (2.9)	
Rural	56 (2.3)	18 (2.5)	19 (1.8)	19 (2.8)	
**Annual income, *n* (%)**					0.001
<$11,999	1213 (49.8)	311 (44.1)	548 (51.5)	354 (53.2)	
$12,000-$49,999	1038 (42.6)	332 (47.1)	429 (40.3)	277 (41.6)	
≥$50,000	184 (7.6)	62 (8.8)	87 (8.2)	35 (5.3)	
**Social Isolation Score**					0.0001
<4	680 (27.0)	242 (32.8)	274 (25.1)	164 (23.9)	
≥4	1835 (73.0)	495 (67.2)	819 (74.9)	521 (76.1)	
**HIV-related factors**					
CD4 cell count, cells/mm^3, median	374 (228, 568)	400 (240, 592)	361 (223, 568)	365 (219, 549)	0.071

(IQR)					
HIV viral load <500 copies/mL, *n* (%)	1690 (67.2)	503 (68.3)	756 (69.2)	431 (62.9)	0.019
VACS Index 2.0 score, median (IQR)	56 (46, 66)	55 (45, 66)	56 (47, 66)	56 (46, 67)	0.066
ART adherent, *n* (%)	1551 (61.7)	479 (65.0)	685 (62.7)	387 (56.5)	0.003
**Other health conditions and status, *n* (%)**					
HCV co-infection	929 (36.9)	202 (27.4)	462 (42.3)	265 (38.7)	<0.0001
Any cancer	524 (20.8)	148 (20.1)	227 (20.8)	149 (21.8)	0.739
Anxiety symptoms	905 (37.1)	219 (30.7)	400 (37.7)	286 (42.9)	<0.0001
Depressive symptoms	534 (21.5)	121 (16.7)	226 (20.8)	187 (27.5)	<0.0001
Pain interference	830 (33.3)	218 (30.0)	364 (33.6)	248 (36.4)	0.035
**Other substance use, *n* (%)**					
Smokes cigarettes	1915 (76.1)	444 (60.2)	893 (81.7)	578 (84.4)	<0.0001
Unhealthy alcohol use	876 (34.8)	207 (28.1)	392 (35.9)	277 (40.4)	<0.0001
Past year stimulants or cocaine	538 (21.4)	77 (10.5)	175 (16.0)	286 (41.8)	<0.0001
**Prescribed opioid receipt, *n* (%)**					0.003
No opioid receipt	1802 (71.7)	559 (75.9)	787 (72.0)	456 (66.6)	
Short-term + low dose	449 (17.9)	108 (14.7)	200 (18.3)	141 (20.6)	
Short-term + high dose	40 (1.6)	12 (1.6)	21 (1.9)	7 (1.0)	
Long-term + low dose	159 (6.3)	43 (5.8)	57 (5.2)	59 (8.6)	
Long-term + high dose	65 (2.6)	15 (2.0)	28 (2.6)	22 (3.2)	
**Prescribed benzodiazepine**					0.521
None	2147 (85.4)	639 (86.7)	934 (85.5)	574 (83.8)	
Low dose	269 (10.7)	70 (9.5)	120 (11.0)	79 (11.5)	
High dose	99 (3.9)	28 (3.8)	39 (3.6)	32 (4.7)	
**Prescribed gabapentin**					0.280
None	2240 (89.1)	668 (90.6)	971 (88.8)	601 (87.7)	
Low dose	127 (5.1)	29 (3.9)	62 (5.7)	36 (5.3)	
High dose	148 (5.9)	40 (5.4)	60 (5.5)	48 (7.0)	
**Prescribed antidepressant**					<0.0001
None	1557 (61.9)	508 (68.9)	653 (59.7)	396 (57.8)	
Short term	314 (12.5)	80 (10.9)	147 (13.5)	87 (12.7)	
Long term	644 (25.6)	149 (20.2)	293 (26.8)	202 (29.5)	
**Site**					0.109
Atlanta	403 (16.0)	141 (19.1)	158 (14.5)	104 (15.2)	
Bronx	258 (10.3)	76 (10.3)	114 (10.4)	68 (9.9)	
Houston	335 (13.3)	99 (13.4)	143 (13.1)	93 (13.6)	
Los Angeles	334 (13.3)	94 (12.8)	131 (12.0)	109 (15.9)	

New York	399 (15.9)	105 (14.3)	190 (17.4)	104 (15.2)	
Baltimore	282 (11.2)	77 (10.5)	137 (12.5)	68 (9.9)	
Washington DC	408 (16.2)	121 (16.2)	181 (16.6)	106 (15.5)	
Pittsburgh	96 (3.8)	24 (3.3)	39 (3.6)	33 (4.8)	
**Calendar year**					0.145
2002–2006	781 (31.5)	245 (33.8)	338 (31.3)	198 (29.3)	
2007–2011	976 (39.3)	275 (37.9)	442 (40.9)	259 (38.3)	
2012–2017	724 (29.2)	205 (28.3)	300 (27.8)	219 (32.4)	
Average follow-up years, mean (SD)	7.0 (3.7)	6.9 (3.7)	7.0 (3.7)	7.3 (3.7)	0.083
Died during study	904 (35.9)	260 (35.3)	406 (37.2)	238 (34.7)	0.534

Table S2. Generalized estimating equation (GEE) analysis for the association between frequency of cannabis use with undetectable HIV viral load status (<500 copies/ml vs. ≥500 copies/ml) among *n*=2,515 PWH engaged in care. Results are presented as odds ratios (OR) from the unadjusted analysis, adjusted for all covariates, and adjusted for all covariates plus adherence to antiretroviral therapy.

	Unadjusted Odds Ratios [95% CI]	Model I, Adjusted Odds Ratios [95% CI]	Model II, Adjusted Odds Ratios [95% CI]	Model III, Adjusted Odds Ratios [95% CI]	Model IV, Adjusted Odds Ratios [95% CI]
**Cannabis**					
No lifetime	Ref	Ref	Ref	Ref	Ref
Lifetime but none in past year	0.95 (0.84–1.08)	0.91 (0.80–1.04)	0.88 (0.77–0.99)	0.95 (0.79–1.15)	0.96 (0.79–1.16)
Past year use	**1.17 (1.01–1.36)**	1.04 (0.90–1.22)	0.89 (0.76–1.05)	0.99 (0.80–1.24)	0.98 (0.78–1.23)
**Age**		**0.95 (0.94, 0.95)**	**0.95 (0.94–0.95)**	**0.96 (0.95–0.97)**	**0.96 (0.95–0.97)**
**Race/ethnicity**					
Non-Hispanic White		Ref	Ref	Ref	Ref
Non-Hispanic Black		**1.21 (1.02–1.43)**	1.15 (0.97–1.36)	1.20 (0.96–1.50)	1.12 (0.89–1.40)
Hispanic (any race)		0.84 (0.65–1.09)	0.82 (0.63–1.06)	0.93 (0.67–1.29)	0.93 (0.66–1.30)
Other (multiple race or unknown)		1.00 (0.69–1.44)	0.96 (0.66–1.40)	1.19 (0.76–1.86)	1.14 (0.72–1.79)
**Sex**					
Male		Ref	Ref	Ref	Ref
Female		**0.62 (0.40–0.94)**	**0.62 (0.41–0.95)**	0.64 (0.36–1.14)	**0.56 (0.32–0.97)**
**Unhealthy alcohol use**					
Yes			**1.15 (1.01–1.30)**	**0.83 (0.70–0.98)**	**0.78 (0.66–0.93)**
No			Ref	Ref	Ref
**Past-year stimulant/cocaine use**					
Yes			**1.58 (1.37–1.81)**	**1.45 (1.18–1.79)**	**1.37 (1.11–1.69)**
No			Ref	Ref	Ref
**Opioid Use**					
No opioid receipt			Ref	Ref	Ref
Short-term + low dose			1.02 (0.90–1.15)	0.98 (0.81–1.20)	0.97 (0.80–1.19)
Short-term + high dose			1.04 (0.67–1.61)	1.86 (0.99–3.49)	1.72 (0.92–3.23)
Long-term + low dose			1.22 (0.99–1.49)	1.27 (0.93–1.73)	1.24 (0.90–1.72)
Long-term + high dose			0.98 (0.70–1.36)	0.82 (0.50–1.35)	0.85 (0.52–1.40)
**Marial Status**					
Never married				Ref	Ref
Married/live with a partner				1.12 (0.88–1.43)	1.10 (0.86–1.42)
Divorced/widowed				1.03 (0.80–1.33)	1.01 (0.78–1.30)
**Homeless**					
Yes				1.02 (0.84–1.22)	0.98 (0.82–1.18)
No				Ref	Ref
**Income**					
<$11,999				Ref	Ref
$12,000-$49,999				1.08 (0.90–1.30)	1.07 (0.89–1.29)
>=$50,000				1.04 (0.74–1.47)	1.05 (0.75–1.49)
**Social score**					
<4				Ref	Ref
>=4				1.01 (0.81–1.26)	1.03 (0.82–1.29)
**HCV**					
Yes				0.98 (0.82–1.18)	0.94 (0.78–1.12)
No				Ref	Ref
**Anxiety**					
Yes				1.04 (0.86–1.27)	1.02 (0.84–1.24)

No				Ref	Ref
**Depression**					
Yes				1.13 (0.92–1.40)	1.07 (0.86–1.32)
No				Ref	Ref
**Smoking**					
Yes				0.82 (0.66–1.00)	0.83 (0.68–1.02)
No				Ref	Ref
**Antidepressant**					
None				Ref	Ref
Short-term				1.24 (0.97–1.58)	1.23 (0.96–1.58)
Long-term				1.13 (0.92–1.38)	**1.27 (1.03–1.55)**
**ART adherence**					
Yes					**0.41 (0.35–0.48)**
No					Ref

Bold indicates statistically significant results.

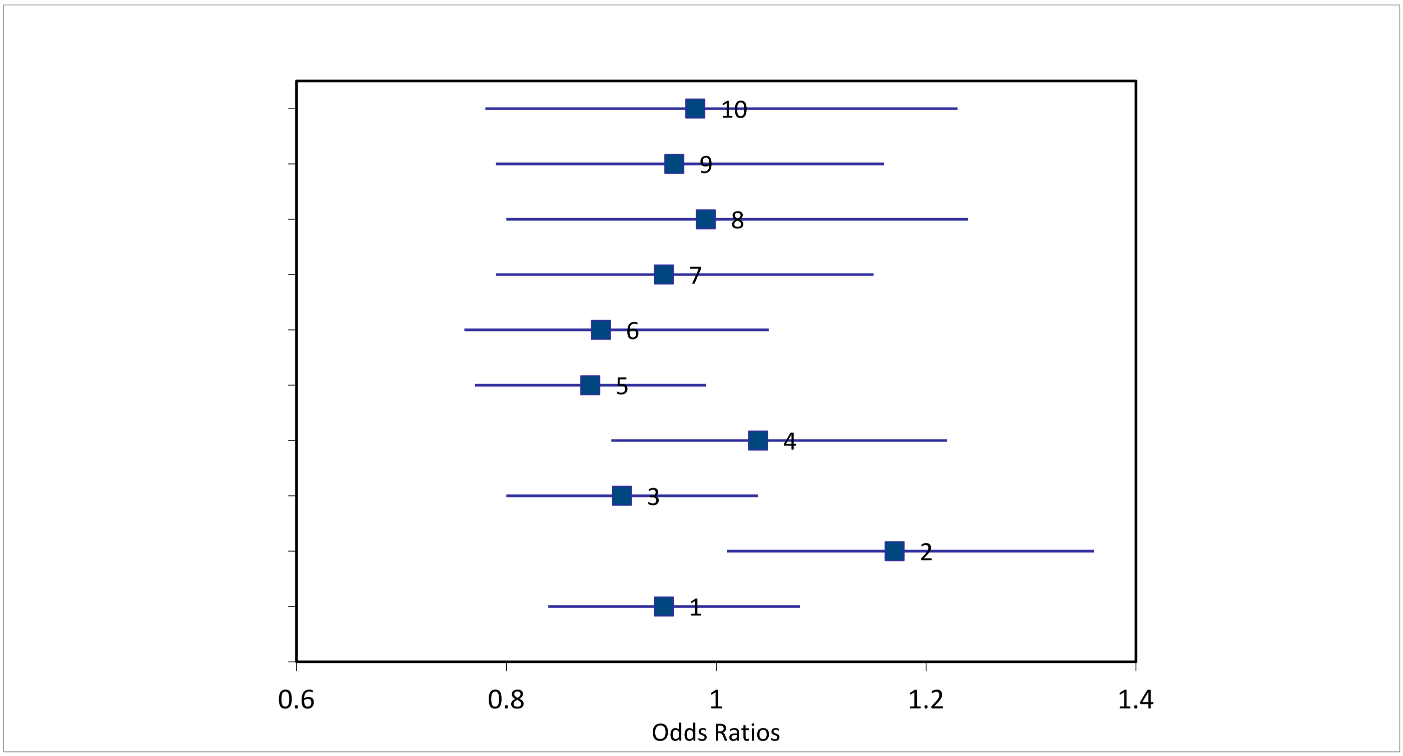

Variable	Cannabis Use	Odds Ratio	Lower 95%CI	Upper 95%CI	Lower Bound	Upper Bound	Order
Lifetime, Unadjusted Odds Ratios [95% CI]	Lifetime but no past-year use	0.95	0.84	1.08	0.11	0.13	1
	Past-year	1.17	1.01	1.36	0.16	0.19	2
Model I, Adjusted Odds Ratios [95% CI]	Lifetime but no past-year use	0.91	0.80	1.04	0.11	0.13	3
	Past-year	1.04	0.90	1.22	0.14	0.18	4
Model II, Adjusted Odds Ratios [95% CI]	Lifetime but no past-year use	0.88	0.77	0.99	0.11	0.11	5
	Past-year	0.89	0.76	1.05	0.13	0.16	6
Model III, Adjusted Odds Ratios [95% CI]	Lifetime but no past-year use	0.95	0.79	1.15	0.16	0.20	7
	Past-year	0.99	0.80	1.24	0.19	0.25	8
Model IV, Adjusted Odds Ratios [95% CI]	Lifetime but no past-year use	0.96	0.79	1.16	0.17	0.20	9
	Past-year	0.98	0.78	1.23	0.2	0.25	10

Figure S1. Forest Plot for Odds Ratios on the Association Between Cannabis Use & Detectable HIV Viral Load for Supplementary Table S2.
